# Identification of monotonically classifying pairs of genes for ordinal disease outcomes

**DOI:** 10.1093/bioadv/vbag143

**Published:** 2026-05-22

**Authors:** Océane Fourquet, Daria Afenteva, Kaiyang Zhang, Sampsa Hautaniemi, Martin S Krejca, Carola Doerr, Benno Schwikowski

**Affiliations:** Computational Systems Biomedicine Lab, Institut Pasteur, Université Paris Cité, Paris 75015, France; LIP6, CNRS, Sorbonne Université, Paris 75005, France; Research Program in Systems Oncology, Research Programs Unit, Faculty of Medicine, University of Helsinki, Helsinki 00014, Finland; Research Program in Systems Oncology, Research Programs Unit, Faculty of Medicine, University of Helsinki, Helsinki 00014, Finland; Department of Pathology, University of Helsinki and HUS Diagnostic Center, Helsinki University Hospital, Helsinki 00014, Finland; Research Program in Systems Oncology, Research Programs Unit, Faculty of Medicine, University of Helsinki, Helsinki 00014, Finland; LIX, CNRS, École Polytechnique, Institut Polytechnique de Paris, Honoré d’Estienne d’Orves, Palaiseau 91120, France; LIP6, CNRS, Sorbonne Université, Paris 75005, France; Computational Systems Biomedicine Lab, Institut Pasteur, Université Paris Cité, Paris 75015, France

## Abstract

**Summary:**

We extend an existing classification method for identifying pairs of genes whose joint expression is associated with binary outcomes to ordinal multi-class outcomes, such as overall survival or disease progression. Our approach, called **multi-class bivariate monotonic classifiers (MBMC)**, is motivated by the need for interpretable classifiers that can provide insights into the underlying biological mechanisms. It can be easily adapted to different research questions, such as identifying gene pair signatures or functional enrichment. We demonstrate that our method is comparable to state-of-the-art classification approaches in terms of performance while offering the benefit of higher interpretability and adaptability to solve different research questions. Our evaluation on three real-world use cases in glioblastoma, high-grade serous ovarian carcinoma, and breast cancer shows that our approach can effectively predict ordinal outcomes and provide novel biological insights.

**Availability and implementation:**

The code is available at https://github.com/oceanefrqt/MBMC.

## 1 Introduction

Advances in transcriptomic profiling, particularly RNA sequencing (RNAseq), have revolutionized the use of gene expression and its role in the study of complex diseases such as cancer. RNAseq provides a comprehensive view of the transcriptome, enabling the identification of differentially expressed genes (DEGs) associated with various cancer-related phenotypes, such as tumor grade, disease progression, or overall survival. Differential expression analysis, where gene expression levels are compared between conditions or groups, is widely used to pinpoint genes that may drive oncogenesis or serve as biomarkers for clinical outcomes. Among DEGs, monotonically DEGs (MDEGs; Wang *et al*. 2015), whose expression levels consistently increase or decrease across an ordered set of clinical states, such as tumor grades or stages, are of particular interest (Stroggilos *et al.* 2022). The identification of MDEGs provides hypotheses about genes that are individually related to cancer progression, offering potential targets for diagnosis and therapy (Tian 2019).

Many studies rely on the assumption that high or low levels of potential biomarkers are associated with a risk of disease or any other relevant outcome. This assumption is restrictive and can prevent the identification of certain other types of relationships between biomarkers and outcomes, such as U-shaped relationships, where both low and high levels of a biomarker are associated with an increased risk of disease. However, in many cases, the existence of genes that are monotonically related to an outcome provides a reasonable working assumption for data-driven learning, especially when both data and prior knowledge about the relationship between gene expression and the outcome are limited (Ghosh 2007, Conde *et al.* 2021).

Note that general monotonic classifiers contain a venerable workhorse of statistical learning: any linear classifier whose weights are fixed in sign (e.g. the non-negative-weight versions of logistic regression, perceptron, or linear support vector machine; SVM) is itself monotone because the decision function is an affine map that preserves the coordinate-wise ordering of the input. Thus, adopting monotonicity does not exclude the traditional linear modeling paradigm but rather subsumes it while still permitting richer non-parametric monotone relationships beyond simple hyperplanes.

Although identifying DEGs and MDEGs provides valuable insight into gene expression patterns, these approaches treat genes independently and, therefore, cannot capture higher-order interactions that underlie biological processes. For example, in certain glioblastomas, the interaction between *TP53* (a tumor suppressor) and MDM2 (an oncogene that inhibits *TP53*) is a critical factor for tumor progression. Although *TP53* and MDM2 can be identified as DEGs, their individual gene expression does not capture this interaction. To overcome this modeling limitation, approaches such as top-scoring pairs (TSP), developed by Frank and Hall (2001), use simple linear decision rules based on pairs of gene expressions.

Nikolayeva *et al.* (2018) extended this approach by identifying monotonic relationships between the expression of two genes and a binary outcome, relying on bivariate monotonic classifiers (BMCs). However, clinical outcomes are often not binary, but on a more diverse discrete scale, i.e. tumor grade, disease progression, or patient survival. Predicting these outcomes is an ordinal classification problem, distinct from the more common categorical classification. In other words, ordinal classification algorithms aim to respect the ordered nature of the outcome, ensuring that predicted categories follow the inherent ranking, making them particularly suited for cancer-related outcomes (Chen 2012).

In this paper, we provide a new method for building ordinal **multi-class bivariate monotonic classifiers (MBMC)**, an extension of the classifiers described by Nikolayeva *et al.* (2018), and to identify pairs of genes of interest. MBMC is primarily intended as an interpretable gene-pair discovery and screening framework for ordinal outcomes. It can also serve as a lightweight predictive model, and as an interpretable complement to higher-capacity predictors when mechanistic insight is desired or limited data is available for training, as common in many biomedical applications. We therefore demonstrate the potential of our approach to advance the understanding of the molecular mechanisms underlying cancer.

## 2 Methods

This section describes the datasets we used for our empirical evaluation (Section 2.1), followed by a technical description of the methodologies (Section 2.2). It includes a formal description of the ordinal classification setting (Section 2.2.1), a detailed description of the new MBMC approach (Section 2.2.3), and the state-of-the-art classification methods (Section 2.2.2) we use for comparison. Section 2.4 provides our interpretation of the results.

### 2.1 Datasets

We selected three transcriptomic datasets that focus on cancer types with well-defined ordinal outcomes. After normalization (see Supplementary Material, available as supplementary data at *Bioinformatics Advances* online, for platform-specific details), genes were filtered using the median absolute deviation (MAD) technique (Howell 2005) to reduce dimensionality and improve signal-to-noise ratio by retaining only highly variable genes across samples. We acknowledge that MAD filtering, while essential for dimensionality reduction, may exclude genes with low cross-sample variability that nonetheless have clinical significance. MAD-specific thresholds for each dataset are provided in the Supplementary Material, available as supplementary data at *Bioinformatics Advances* online. Each dataset was then divided into training (80%) and testing (20%) subsets using stratified sampling to preserve class proportions.

#### 2.1.1 Glioblastoma dataset

This microarray-based dataset comprises primary tumor samples from 70 glioblastoma patients collected by the German Glioma Network (Reifenberger *et al.* 2014). The patients are divided into 23 long-term (>36 months overall survival), 16 short-term (<12 months), and 31 intermediate survivors. The raw data are available on the NCBI Gene Expression Omnibus (https://www.ncbi.nlm.nih.gov/geo/, accession GSE53733). The final analysis included 1836 genes (see Supplementary Material, available as supplementary data at *Bioinformatics Advances* online, for preprocessing). The numbers of patients per class for training and testing are summarized in Table 1.

**Table 1:** Class balance in glioblastoma training and testing datasets.^a^

	Short-term survivors	Intermediate-term survivors	Long-term survivors
Training data	13	25	18
Testing data	3	6	5

a The numbers represent the number of primary tumor samples from the glioblastoma patients in each category.

#### 2.1.2 Ovarian high-grade serous carcinoma dataset

Bulk RNAseq data from the DECIDER project (https://www.deciderproject.eu) are used to study high-grade serous ovarian carcinoma (Lahtinen *et al.* 2023). The cohort includes primary tumors, intra-abdominal lesions, and ascites from patients undergoing surgery followed by platinum-based chemotherapy (https://clinicaltrials.gov/study/NCT04846933). Cancer-specific expression profiles are obtained using the PRISM deconvolution algorithm (Hakkinen *et al.* 2021). Patients are classified according to their platinum-free interval (PFI): resistant (<6 months), semi-sensitive (6–12 months), or sensitive (>12 months; Luyckx *et al.* 2022). The numbers of genes and class distributions are summarized in Table 2. Full preprocessing details are provided in the Supplementary Material, available as supplementary data at *Bioinformatics Advances* online.

**Table 2:** Class balance in training and testing datasets.^a^

Sample types	Data	Platinum-resistant	Semi-sensitive	Platinum-sensitive
Primary tumors	Training	28	34	64
(1126 genes)	Testing	7	9	16
Ascites	Training	34	22	26
(1295 genes)	Testing	9	6	6
Intra-abdominal	Training	58	34	62
(1115 genes)	Testing	14	9	16

a The numbers represent the number of samples in each category.

#### 2.1.3 Breast cancer dataset

The METABRIC cohort (Curtis *et al.* 2012, Pereira *et al.* 2016) includes genomic and transcriptomic data from more than 2000 breast cancer patients (https://www.cbioportal.org/study/summary?id=brca_metabric). We focus on predicting relapse-free survival (RFS) using transcriptomic profiles. Only patients who experienced recurrence (n=630) were included. The RFS values were divided into three equally sized ordinal classes. After gene filtering (see Supplementary Material, available as supplementary data at *Bioinformatics Advances* online), 1708 genes were retained. Both training and testing breast cancer datasets included the same numbers of samples in relapse categories. Each category—short-term, intermediate-term, and long-term relapse—contained 105 tumor samples.

### 2.2 Algorithmic framework

This section defines the concept of ordinal classification (Section 2.2.1), introduces our new approach for multi-class ordinal monotone classification (Section 2.2.3), and summarizes the evaluation metrics and the algorithms to which the method is compared (Section 2.2.2).

#### 2.2.1 Ordinal classification

Ordinal classification (Gutiérrez and García 2016) corresponds to the task of predicting an outcome with ordered categories such as *low*, *medium*, and *high*. Its key characteristic is that it takes into account the **relative ranking** of these categories. For example, we know that *low* is worse than *medium*, and *medium* is worse than *high*.

Monotonic classification is a subtype of the ordinal classification task in which monotonicity constraints are added, meaning that the learned relationship between input features and the outcome is monotonic. A classifier *f* is called **monotonic** if, for any i∈{1,…,n} and any Δ∈R, the sign of $f(\ldots ,x_{i},\ldots )-f(\ldots ,x_{i}+\Delta ,\ldots )$ does not take on both −1 and 1 over the domain $(x_{1},\ldots ,x_{n})\in R^{n}$.

Ordinal classification performance can be assessed using several standard metrics (Gaudette and Japkowicz 2009, Cardoso and Sousa 2011). In this study, we report results of mean absolute error (MAE), accuracy (*Acc*), Cohen’s Kappa (κ), and the Matthews correlation coefficient (MCC). These metrics capture complementary aspects of predictive performance (see Supplementary Material, available as supplementary data at *Bioinformatics Advances* online, for detailed definitions and additional metrics). Among them, MAE is particularly well suited for ordinal data (Gaudette and Japkowicz 2009), as it naturally accounts for the ordered nature of class labels. To ensure consistency, we assume equal spacing (distance of 1) between consecutive classes in all experiments.

#### 2.2.2 Other commonly used classifiers

The **scikit-learn** library (Pedregosa *et al.* 2011) offers numerous implementations of classic classification algorithms, such as **decision trees** (*DT*), **random forests** (*RF*), **logistic regression** (*LR*), **Gaussian processes** (*GP*), and **support vector machines with linear and radial basis function kernels** ($SVM_{\text{linear}}$ and $SVM_{\text{rbf}}$). However, in the versions provided by scikit-learn, the algorithms typically treat each class as categorical and independent, thus failing to exploit the information contained in the ordering of the classes (in the case of ordinal classification). For non-monotone baselines, we used the widely adopted k−1 reduction to binary tasks, which incorporates the class ordering through ordered thresholds but does not impose monotonicity and does not explicitly model unequal ordinal distances (Frank and Hall 2001). We interpret these baselines as pragmatic comparators to widely-used baselines rather than fully ordinal-optimized methods, such as cumulative link models or ordinal SVM-type approaches. The GridSearch method from scikit-learn was used to systematically explore a predefined range of hyperparameters and identify the best combination of parameters for each model.

#### 2.2.3 Multi-class bivariate monotonic classifier

BMCs can be constructed using an efficient dynamic-programming regression algorithm Stout (2013). Nikolayeva *et al.* (2018) extended this framework by introducing an ensemble classifier, ensembleBMC, to predict binary disease outcomes from transcriptomic data. In this work, we generalize ensembleBMC to the multi-class setting, introducing **ensembleMBMC**, which is built upon a multi-class extension of the BMC that we term the **MBMC**.

To illustrate the operation of the **MBMC**, consider a dataset with *n* samples. Each sample belongs to one of *p* ordered classes, with class labels ranging from 1 to *p* (where p<n). The goal is to find a set of monotonic functions that separate these ordered classes, minimizing the $L_{1}$-error.

The MBMC is based on the same regression algorithm as traditional BMC (Stout 2013), which uses a divide-and-conquer strategy to divide any regression problem with more than two classes into two subproblems when needed, to afterwards merge the resulting solutions. It consists of recursively dividing the classes into subgroups (see the Supplementary Material, available as supplementary data at *Bioinformatics Advances* online, for an illustration). Initially, all classes are grouped together. We then split them into two subgroups: one containing classes 1 to *k* and the other containing classes (k+1) to *p*. Here, *k* is often chosen as the integer part of p/2. Stout (2013) proves that the separation function between these two subgroups can be found in O(n log n) time complexity. Following this initial separation, the process can be recursively applied to the resulting subgroups. Each subgroup is further divided into two based on class labels, and a separation function is determined for the new subgroups. This recursive process continues until a separation function is found for each pair of classes. While the complexity of finding a single separation function is O(n log n), the overall complexity of recursively separating all class pairs is likely closer to O(np log n) in the worst case, as class sizes can be imbalanced. The approach requires ordered classes.

Based on the regression algorithm above, the MBMC determines the pairs of top-performing gene expressions (for reasons of brevity, from now on, we refer to pairs of genes instead of pairs of gene expression values) through *k*-fold cross-validation.

Because candidate gene pairs are ranked by cross-validated error under a monotone model, the selected top pairs are, by construction, those best explained by a monotone relationship; pairs exhibiting strongly non-monotone behavior are automatically discarded during model selection.

Since a simple brute-force method for calculating the performance of all existing gene pairs in the dataset is both time consuming and memory intensive, we instead apply a preselection algorithm that acts as a heuristic to determine pairs that perform well early on (Fourquet *et al.* 2025). This preselection identifies and selects the pairs for which the cross-validation is calculated, based on the idea that calculating the MAE on the whole dataset (${\text{MAE}}_{\text{full}}$) gives a lower bound on the MAE calculated with cross-validation (${\text{MAE}}_{\text{CV}}$). The algorithm takes as a parameter the minimum number of disjoint pairs, i.e. pairs without genes in common. The details of the algorithm and a mathematical proof of the relationship between both types of MAE are in the Supplementary Material, available as supplementary data at *Bioinformatics Advances* online.

We note that the number of gene pairs required can vary depending on the specific research question. For instance, identifying a gene signature may only require a small set of 5–10 disjoint pairs, whereas functional enrichment analysis typically benefits from a larger set of 50–200 genes, corresponding to 25–100 disjoint pairs. However, constructing and analyzing gene networks may require a set of more than 100 genes. This flexibility in scale is a key advantage of our approach, as it can be tailored to accommodate different research objectives.

An **ensemble model (ensembleMBMC)** is constructed using the method described by Nikolayeva *et al.* (2018), which involves determining the optimal number of gene pairs for an ensemble model and then selecting the top pairs with the constraint that no pair shares a common gene. For a given input, each pair produces a prediction and a majority vote over the discrete class labels produced by each classifier is used to obtain the prediction in the output of the ensembleMBMC. In case of tie, the prediction is biased toward the worst outcome.

### 2.3 Uncertainty estimation

We estimated the variability in the performance estimates via a training bootstrap with a fixed test set. On a fixed random 80/20 train/test split, we generated 1000 bootstrap resamples of the training data; within each resample, we re-selected MBMC pairs, re-tuned any model hyperparameters, trained the model, and evaluated on the held-out test set. We report the median and 95% percentile confidence interval of all test-set metrics across resamples. As the test set was fixed, the confidence intervals reflect training-sample variability. The estimated performance when training on the entire training dataset, along with the ranking, is available in the Supplementary Material, available as supplementary data at *Bioinformatics Advances* online.

### 2.4 Interpretation

The identification of top-performing pairs is interesting from both an individual and a group perspective. Individually, each pair of MBMC relates two genes, capturing their coordinated behavior—whether their expressions rise or fall together or vary inversely between phenotypes. The MBMCs balance simplicity and interpretability, identifying gene pairs with robust, easily understood monotonic patterns. By avoiding the rigidity of linear models and the opacity of complex ones, their bi-dimensional nature offers a nuanced view of molecular relationships. When considered collectively, these pairs can be aggregated into gene signatures or further analyzed through pathway enrichment, as detailed in the Supplementary Material, available as supplementary data at *Bioinformatics Advances* online.

## 3 Empirical evaluation

This section presents the main results of the empirical evaluation on the different datasets presented in Section 2.1. For datasets on high-grade serous carcinoma and glioblastoma and ovarian cancer, it includes a visualization of the best pairs and a comparison with other methods in terms of performance and interpretability. The best performing MBMCs are selected using the algorithm described in the Supplementary Material, available as supplementary data at *Bioinformatics Advances* online, according to three distinct scenarios, with the minimum number of disjoint pairs serving as the variable parameter to identify the best performing pairs. This parameter is set to 5, 10, and 20 in each scenario, and the scenarios are, respectively, labeled as MBMC-5, MBMC-10, and MBMC-20. Each classifier is trained and tuned on the training dataset, using a five-fold cross-validation, and each performance is computed on the testing datasets. For the breast cancer dataset, the analysis consists in a validation of the top-performing pairs using Kaplan–Meier curves and log rank-test on the predictions of the testing data. More detailed analysis, including complete tables with measurement intervals, pathway-based evaluations, and visual comparisons for interpretability, is provided in the Supplementary Material, available as supplementary data at *Bioinformatics Advances* online.

### 3.1 Results on glioblastoma dataset

Table 3 provides the number of identified top pairs and the number of different genes between these pairs for the three scenarios.

**Table 3:** Summary of the number of top pairs and genes identified in the three scenarios for the glioblastoma dataset.

	MBMC-5	MBMC-10	MBMC-20
Number of top pairs	7	23	102
Number of genes among the top pairs	12	35	112

**Figure 1:**
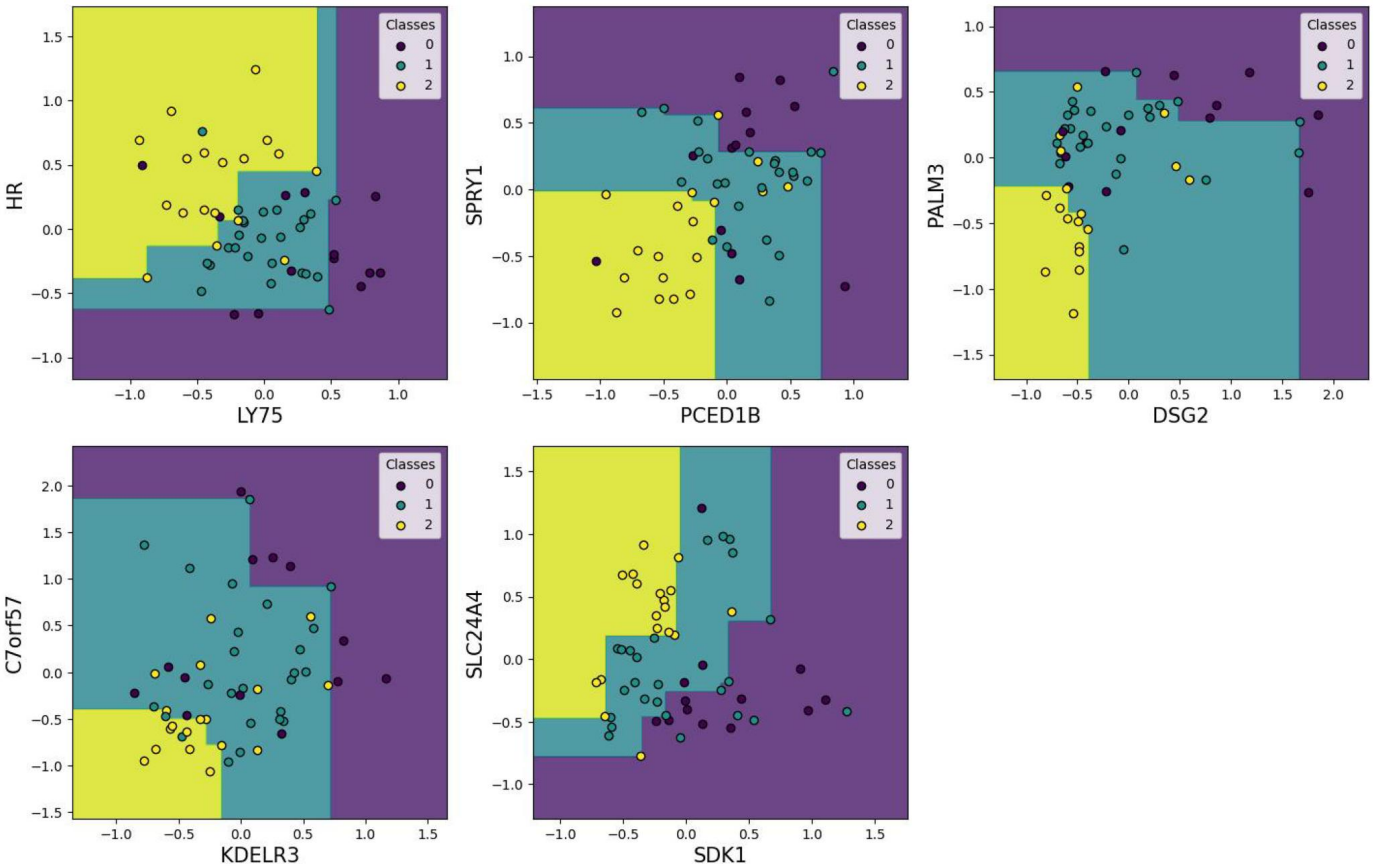
Five non-redundant gene pairs chosen for the MBMC ensemble model, predicting overall survival for glioblastoma patients.

As the MBMC method produces a set of top-pair classifiers, the performance was calculated for the testing data for each classifier, built with the training data. To obtain a single score per metric for each scenario, the median of the performances across the top pairs was taken. Moreover, we computed an ensembleMBMC of five MBMCs (see Fig. 1). Competing classification algorithms (listed in Section 2.2.2), trained on all the genes, were fine-tuned with grid search and adjusted to respect ordinal constraints. Since RF and decision tree algorithms incorporate randomness, we evaluated the median performance of 10 runs.

Across the metrics evaluated (Table 4), the algorithms achieving the strongest overall performance in the glioblastoma survival classification task were LR, SVM (both linear and RBF kernels), RF, and the ensembleMBMC approach. These methods consistently reached median accuracies, exhibited the lowest MAE values. The bootstrap confidence intervals of these methods overlap substantially, indicating that performance differences are small in practical terms; we therefore characterize ensembleMBMC as competitive rather than clearly superior. In contrast, the Gaussian Process classifier performed at chance level across all metrics, while Decision Trees and the smaller MBMC scenarios (MBMC-5 and MBMC-10) showed weaker and more variable results. *GP* performance near chance is expected in the small-sample/high-dimensional regime of transcriptomics, where standard kernels can be poorly matched and the model may over-regularize despite tuning; we therefore treat GP as a reference baseline rather than a best-in-class method in this setting. Although MBMC-20 demonstrated greater robustness, with tighter interquartile ranges, its overall predictive performance remained inferior to that of the best performing algorithms. Although not more effective than other methods, ensembleMBMC achieves performance competitive with the strongest baselines, allows us to study certain gene pairs (Fig. 1).

**Table 4:** Performance evaluation of different models on the glioblastoma dataset.^a^

	MBMC-5	MBMC-10	MBMC-20	ensembleMBMC	RF	DT	LR	GP	**SVM** ${\;}_{\text{linear}}$	**SVM** ${\;}_{\text{rbf}}$
Acc	0.43	0.43	0.43	0.50	0.50	0.43	0.50	0.21	0.50	0.50
	(0.29–0.50)	(0.36–0.50)	(0.43–0.50)	(0.21–0.64)	(0.43–0.61)	(0.29–0.64)	(0.43–0.64)	(0.21–0.21)	(0.29–0.71)	(0.43–0.57)
MAE	0.64	0.71	0.68	0.64	0.57	0.68	0.50	1.14	0.64	0.57
	(0.57–1.00)	(0.57–0.86)	(0.64–0.71)	(0.43–0.86)	(0.43–0.68)	(0.43–0.93)	(0.36–0.71)	(1.14–1.14)	(0.36–0.93)	(0.43–0.71)
MCC	0.12	0.14	0.21	0.24	0.22	0.16	0.26	0.00	0.28	0.21
	(0.00–0.26)	(0.06–0.22)	(0.14–0.22)	(−0.09 to 0.49)	(0.03–0.41)	(−0.14 to 0.48)	(0.05–0.50)	(0.00–0.00)	(−0.06 to 0.61)	(0.00–0.40)
CK	0.11	0.11	0.16	0.19	0.16	0.15	0.20	0.00	0.25	0.13
	(0.00–0.21)	(0.05–0.17)	(0.11–0.20)	(−0.05 to 0.43)	(0.03–0.35)	(−0.13 to 0.46)	(0.03–0.43)	(0.00–0.00)	(−0.05 to 0.57)	(0.00–0.33)

a Metrics include accuracy (Acc), mean absolute error (MAE), Matthews correlation coefficient (MCC), and Cohen’s kappa (CK). Values are reported as median (5%–95% percentile confidence interval).

However, the results obtained in Table 4 should be interpreted with caution, as the short-term survivor class contains only three test samples. At this scale, a single misclassification shifts per-class recall by approximately 33 percentage points, making point estimates unreliable and highly sensitive to individual cases (see Supplementary Material, available as supplementary data at *Bioinformatics Advances* online, for confusion matrix). We acknowledge that bootstrap resampling of the test set, while reported, does not fully mitigate this instability: when the stratum size is n=3, many bootstrap replicates contain only one or two unique observations from that class, meaning the resulting confidence intervals reflect resampling variance rather than true generalization uncertainty. To account for class imbalance across datasets, we compute macro-averaged F1 and balanced accuracy, as these weight each class equally regardless of support (Balanced Accuracy=0.57±[0.33, 0.80], Macro F1=0.54±[0.27, 0.79]). Nevertheless, no resampling procedure substitutes for a larger, prospectively collected cohort. The results on the glioblastoma dataset should therefore be interpreted as exploratory and hypothesis-generating rather than confirmatory, and external validation on an independent cohort remains necessary before clinical conclusions can be drawn.

To pursue exploration, we examine the genes and pairs of the ensembleMBMC. Table 5 summarizes the genes, their roles, and whether they are known as biomarkers in glioblastoma based on a comprehensive review of the literature. Our focus has been on studies that highlight these genes as biomarkers for overall survival, proliferation, and other relevant factors. Although we have done our best to be precise, we cannot guarantee that all pertinent research papers have been included, especially for genes where clear biomarker-related studies were not immediately apparent.

**Table 5:** Genes constituting the ensembleMBMC for the glioblastoma dataset with their role and whether they are already known biomarkers for overall survival, according to a screening of the literature.

Gene	Description	Known biomarker for glioblastoma
*HR*	Involved in hair growth and encodes a transcriptional corepressor for nuclear receptors, including thyroid hormone and vitamin D receptors	No
*LY75*	Plays a role in regulating immune responses and inflammatory response	No
*PCED1B*	Encodes a member of the GDSL/SGNH-like acyl-esterase family, which functions in modifying biopolymers on the cell surface through hydrolysis; multiple splice variants	Yes [cell proliferation and tumorigenesis in Yao *et al.* (2020)]
*SPRY1*	Involved in negative regulation of fibroblast growth factor receptor signaling pathway	Associated with vascular programs enriched in *IDH*-wildtype gliomas (Zhang *et al.*, 2018)
*DSG2*	Plays a role in cell-cell adhesion, particularly in heart and muscle tissues, contributing to tissue integrity and function	No
*PALM3*	Involved in neuron-specific signaling and synaptic transmission, particularly by palmitoylating neural proteins	No
*KDELR3*	Plays a role in retrograde transport of misfolded proteins from the Golgi apparatus back to the endoplasmic reticulum for proper folding	Yes. Overall survival: Feng *et al.* (2024); linked with *IDH* status: Feng *et al.* (2024)
*C7orf57*	Plays a role in cellular metabolic processes and is involved in the regulation of mitochondrial function, although its exact functions are not fully understood	No
*SDK1*	Plays a role in cell to cell communication, embryonic development, angiogenesis, formation of new blood vessels	Yes [diagnostic in Wolter *et al.* (2022)]
*SLC24A4*	Plays a role in transporting ions across cellular membranes	Yes [immune response in Wu *et al.* (2024)]

Among the genes listed, several have known roles in critical cellular processes such as cell adhesion, signaling, and metabolic regulation. *PCED1B*, *SPRY1*, *KDELR3*, *SDK1*, and *SLC24A4* are already established biomarkers, with *PCED1B* linked to cell proliferation and tumorigenesis, *SPRY1* correlated with overall survival, *KDELR3* identified as a prognostic indicator for survival outcomes, *SDK1* utilized in diagnostic contexts, and *SLC24A4* associated with immune response dynamics within the tumor microenvironment. However, genes such as *HR*, *LY75*, *DSG2*, *PALM3*, and *C7orf57* have not yet been well-documented as biomarkers in glioblastoma, although their involvement in key cellular and immune regulatory pathways makes them promising candidates for future research. It is worth noting that the *IDH* genes are absent from our identified genes, despite their central role in distinguishing *IDH*-wildtype from *IDH*-mutant gliomas. This exclusion occurred during MAD filtering due to low expression variability in our cohort, which predominantly consists of *IDH*-wildtype patients (reflecting the 84% prevalence in glioblastoma; Reifenberger *et al.* 2014). Importantly, *Reifenberger et al.* demonstrated that standard differential expression analysis of *IDH*-wildtype glioblastomas did not identify genes distinguishing long-term from short-term survivors, suggesting that survival-associated molecular patterns in this majority subgroup may be captured by higher-order relationships between genes rather than univariate expression differences. For example, the MBMC approach analyzes the full cohort and identifies monotonic relationships between gene pairs that may capture survival-associated patterns across both *IDH*-wildtype and *IDH*-mutant tumors. Moreover, *IDH* status is primarily mutation-defined rather than expression-defined. None of our identified genes correlate with *IDH1* or *IDH2* expression, indicating they capture *IDH*-independent signals.

### 3.2 Results on ovarian high-grade serous carcinoma

The first section compares the results between subtypes, while the second compares them with other classification methods.

#### 3.2.1 Differences between sample types

We analyze the number and composition of the top-ranking gene pairs identified by the MBMC algorithm in the three subtypes of ovarian cancer tissue (primary tumors, ascites, and intra-abdominal lesions). Intra-abdominal samples consistently yielded the highest number of top pairs in all three MBMC settings (MBMC-5, MBMC-10, and MBMC-20), while primary and ascites datasets showed similar but lower counts.

We then examined the overlap of genes between the top pairs from different tissue types. A randomization test revealed that the overlap between primary and intra-abdominal samples was significantly higher than expected by chance ($P=2\times 10^{-5}$), while overlaps involving ascites were not significant, reflecting the distinct biological nature of ascites fluid compared to solid tumor tissues.

To assess the prediction performance, we analyzed the distribution of MAE values across all possible gene pairs for each subtype. The MAE distributions were approximately normal, with intra-abdominal and primary samples showing more concentrated values, while ascites exhibited higher variability. Finally, permutation tests confirmed that the top gene pairs achieved MAE values significantly better than those expected by chance (P<0.05) for all three tissue subtypes.

Detailed methodology, additional statistical analyses, and complete result tables are provided in the Supplementary Material, available as supplementary data at *Bioinformatics Advances* online.

#### 3.2.2 Performance comparison with other algorithms to predict PFI

We compared our MBMC method with standard classification algorithms on the three datasets in Table 6.

**Table 6:** Performance evaluation of different models on the HGSC dataset across the primary sites, intra-abdominal, and ascites subsets.^a^

	MBMC-5	MBMC-10	MBMC-20	ensembleMBMC	RF	DT	LR	GP	**SVM** ${\;}_{\text{linear}}$	**SVM** ${\;}_{\text{rbf}}$
Primary sites										
Acc	0.42	0.41	0.41	0.53	0.52	0.38	0.47	0.22	0.47	0.53
	(0.38–0.47)	(0.36–0.44)	(0.34–0.44)	(0.38–0.66)	(0.44–0.58)	(0.27–0.52)	(0.34–0.56)	(0.22–0.22)	(0.34–0.56)	(0.44–0.59)
MAE	0.70	0.73	0.75	0.59	0.64	0.80	0.69	1.28	0.78	0.69
	(0.67–0.78)	(0.69–0.81)	(0.72–0.81)	(0.47–0.75)	(0.56–0.73)	(0.62–1.00)	(0.53–0.84)	(1.28–1.28)	(0.59–0.94)	(0.53–0.81)
MCC	0.11	0.06	0.05	0.26	0.17	0.05	0.15	0.00	0.11	0.16
	(0.04–0.16)	(0.01–0.11)	(0.01–0.07)	(0.06–0.44)	(0.03–0.30)	(−0.14 to 0.24)	(−0.03 to 0.33)	(0.00–0.00)	(−0.08 to 0.29)	(−0.10 to 0.37)
CK	0.10	0.06	0.04	0.23	0.15	0.05	0.14	0.00	0.11	0.11
	(0.03–0.15)	(0.01–0.10)	(0.01–0.06)	(0.05–0.41)	(0.03–0.27)	(−0.14 to 0.24)	(−0.03 to 0.31)	(0.00–0.00)	(−0.08 to 0.28)	(−0.04 to 0.32)
Intra-abdominal										
Acc	0.31	0.32	0.33	0.28	0.38	0.33	0.33	0.36	0.41	0.44
	(0.28–0.33)	(0.31–0.33)	(0.31–0.33)	(0.21–0.38)	(0.31–0.46)	(0.23–0.45)	(0.23–0.46)	(0.36–0.36)	(0.31–0.51)	(0.33–0.54)
MAE	0.95	0.92	0.92	1.03	0.87	0.90	0.87	1.05	0.92	0.87
	(0.92–1.00)	(0.90–0.95)	(0.90–0.95)	(0.85–1.18)	(0.77–0.99)	(0.73–1.09)	(0.72–1.03)	(1.05–1.05)	(0.74–1.10)	(0.72–1.03)
MCC	−0.04	−0.02	−0.01	−0.09	0.06	0.02	0.03	0.00	0.06	0.12
	(−0.08 to −0.00)	(−0.05 to 0.01)	(−0.03 to 0.01)	(−0.21 to 0.06)	(−0.04 to 0.17)	(−0.14 to 0.19)	(−0.15 to 0.21)	(0.00–0.00)	(−0.11 to 0.23)	(−0.04 to 0.28)
CK	−0.04	−0.02	−0.01	−0.08	0.06	0.02	0.03	0.00	0.06	0.11
	(−0.08 to −0.00)	(−0.05 to 0.01)	(−0.03 to 0.01)	(−0.21 to 0.06)	(−0.04 to 0.16)	(−0.14 to 0.19)	(−0.15 to 0.21)	(0.00–0.00)	(−0.11 to 0.23)	(−0.04 to 0.27)
Ascites										
Acc	0.36	0.38	0.38	0.38	0.36	0.33	0.24	0.43	0.33	0.29
	(0.31–0.43)	(0.33–0.43)	(0.38–0.43)	(0.24–0.52)	(0.24–0.48)	(0.14–0.52)	(0.10–0.38)	(0.43–0.43)	(0.14–0.48)	(0.29–0.48)
MAE	0.79	0.76	0.76	0.71	0.81	0.86	0.95	0.86	1.05	0.71
	(0.71–0.86)	(0.71–0.86)	(0.71–0.81)	(0.57–0.9)	(0.69–1.00)	(0.59–1.14)	(0.76–1.24)	(0.86–0.86)	(0.76–1.33)	(0.62–0.86)
MCC	0.04	0.06	0.09	0.06	0.04	0.00	−0.13	0.00	−0.06	0.00
	(−0.05 to 0.13)	(0.00–0.12)	(0.05–0.14)	(−0.13 to 0.26)	(−0.14 to 0.19)	(−0.28 to 0.28)	(−0.36 to 0.11)	(0.00–0.00)	(−0.33 to 0.20)	(−0.05 to 0.19)
CK	0.04	0.05	0.09	0.06	0.04	0.00	−0.12	0.00	−0.05	0.00
	(−0.04 to 0.12)	(0.00–0.11)	(0.04–0.13)	(−0.13 to 0.26)	(−0.14 to 0.17)	(−0.27 to 0.28)	(−0.34 to 0.10)	(0.00–0.00)	(−0.29 to 0.18)	(−0.03 to 0.13)

a Metrics include accuracy (Acc), mean absolute error (MAE), Matthews correlation coefficient (MCC), and Cohen’s kappa (CK). Values are reported as median (5%–95% percentile confidence interval).

In the primary sites subset, performance varies noticeably across models, with SVM${\;}_{\text{rbf}}$, RFs, and ensembleMBMC forming the top-performing group. Both SVM${\;}_{\text{rbf}}$ and RF achieve high accuracies (≥0.52) with strong MCC and Cohen’s κ values, indicating that they effectively capture the nonlinear and heterogeneous structure of primary tumor gene expression. The MBMC-5/10/20 show limited performance with the increasing numbers of gene pairs and plateau around 0.41 accuracy with low correlation-based metrics, suggesting restricted flexibility. ensembleMBMC, however, substantially improves the framework achieving 0.53 accuracy and the lowest MAE—demonstrating that aggregating MBMC recovers important signal while retaining structural interpretability. This pattern highlights that, while monotonicity constraints alone may be too restrictive, ensemble-based monotonic modeling offers a competitive alternative for this subtype.

The intra-abdominal subtype presents the greatest predictive difficulty, with all models performing near chance and most MCC/Cohen’s κ values hovering around zero or slightly negative. Accuracies span a narrow range (0.28–0.44). Even flexible nonlinear models such as SVM${\;}_{\text{rbf}}$ and GP achieve only modest advantages, and their low MCC values suggest limited class separability. MBMC method perform similarly to weaker baselines, and ensembleMBMC does not provide measurable improvement. In fact, it slightly underperforms the MBMC-5/10/20, likely due to increased smoothing in the ensemble amplifying the already small inter-class differences. Overall, the results underscore that the difficulty arises from intrinsic overlap between classes rather than insufficient model complexity, making this subtype the most challenging for all approaches.

In the ascites subtype, predictive performance improves relative to the Intra-abdominal group, and the MBMC framework becomes considerably more competitive. MBMC-10, MBMC-20, and ensembleMBMC all achieve accuracies around 0.38, with positive MCC and Cohen’s κ values indicating meaningful class discrimination. The ensembleMBMC, in particular, exhibits the lowest MAE among all models in this subtype, suggesting more stable and less error-prone predictions. Although GP reach the highest accuracy (0.43), their correlation metrics near zero imply a tendency toward majority-class prediction rather than genuine class-specific modeling. In contrast, the MBMC models, especially the MBMC-10, the MBMC-20, and the ensembleMBMC, capture classes structures more effectively than linear or tree-based baselines. These results suggest that the ensembleMBMC aligns well with the biological characteristics of ascites samples, making this subtype the most favorable context for MBMC models. As in the case of glioblastoma, however, note that confidence intervals among the best methods tend to significantly overlap, also for other subsets of the data.

### 3.3 Results on breast cancer

In this analysis, we first study the ensembleMBMC obtained and validate it using Kaplan–Meier curves and log-rank tests. We then study the top-performing pairs to see if, individually, we can also validate them.

#### 3.3.1 Identification of a gene pair signature with the ensembleMBMC

**Figure 2:**
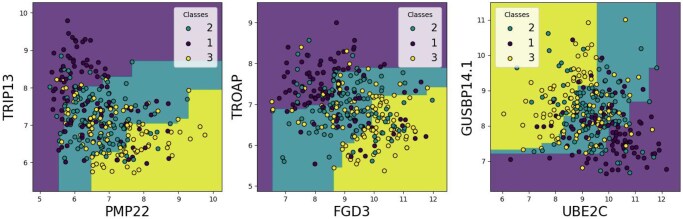
The ensembleMBMC constructed for breast cancer.

As described in Section 3.1, the method developed by Nikolayeva *et al.* (2018) allowed us to build an ensemble model made of MBMCs. The resulting ensembleMBMC contains three MBMC, with an MAE of 0.62 and an MCC of 0.17 on the testing data (Fig. 2).

To validate the interest and significance of this model, we predicted the classes of the testing data and then compared them with Kaplan–Meier curves and log-rank tests, separating the samples according to the predicted class. Kaplan–Meier curves allowed the visual representation of potential differences in relapse between groups, and log-rank tests were used to assess their statistical significance.

**Figure 3:**
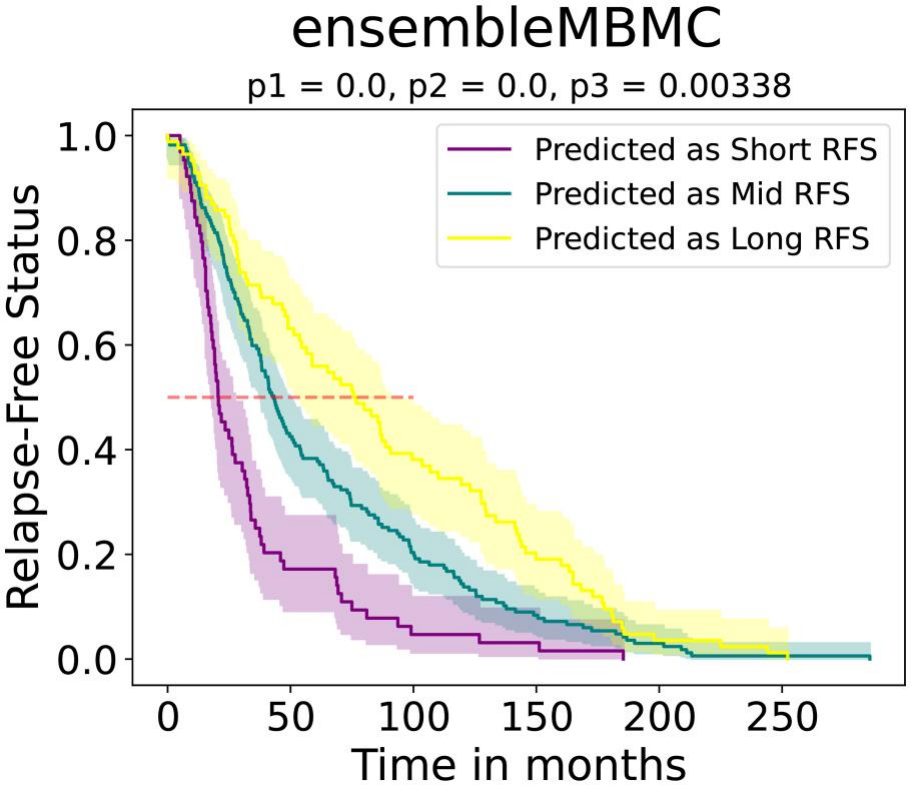
Survival curves for the breast cancer ensembleMBMC, separating patients according to their predicted RFS. The colors are taken from those of the MBMCs visualization. The values of p1, p2, and p3 correspond to the *P*-values of the log-rank tests between short and mid RFS, short and long RFS, and mid and long RFS, respectively. To be significant, *P*-values must be less than α=0.05/3=0.0167.

As shown in Fig. 3, the three predicted groups that exhibited distinct RFS were well-separated and coherent. For example, the median time to relapse with a probability of 0.5 was approximately 25 months for the short RFS group, 50 months for the mid RFS group and 75 months for the long RFS group. This resulted in an approximately 2-year separation between each successive group, a distinction that is of clinical interest and has the potential for further study. Moreover, log-rank tests comparing each group, with a Bonferroni-corrected significance threshold of α=0.05/3=0.0167, revealed significant differences between the three groups. Thus, we conclude that this ensemble of gene pairs effectively predicted whether relapse would occur in the short, mid, or long term. A threshold-sensitivity analysis showed that quantitative performance depends on the chosen class boundaries, with more balanced threshold definitions yielding more informative MCC values than strongly imbalanced ones (Table 11, available as supplementary data at *Bioinformatics Advances* online).

The results obtained on the METABRIC dataset in Table 7 indicate that the overall performance of the model remains relatively modest, reflecting the intrinsic difficulty of the classification task. Among all evaluated methods, the best-performing models are ensembleMBMC, LR, and the SVMs, particularly with the RBF kernel, which achieves the highest accuracy and the strongest global agreement metrics. RF also delivers intermediate and stable performance, slightly below. In contrast, the Decision Tree and, most notably, the Gaussian Process classifier show clearly inferior results, with MCC and Cohen’s κ values near zero, indicating near-random behavior for GP and limited robustness for DT. The GP classifier yielding constant predictions across all cross-validation runs on the METABRIC dataset, reflected by zero-width confidence intervals, might indicate that the model collapsed to a degenerate solution (likely due to the high dimensionality of the feature space relative to the kernel’s ability to generalize). The MAE values follow the same pattern as the other metrics: SVMs, LR, and ensembleMBMC display better calibration with lower errors, whereas DT and GP produce substantially higher MAE.

**Table 7:** Performance of different models on the breast cancer dataset.^a^

	ensembleMBMC	RF	DT	LR	GP	**SVM** ${\;}_{\text{linear}}$	**SVM** ${\;}_{\text{rbf}}$
**Acc**	0.43	0.45	0.38	0.44	0.33	0.42	0.46
	(0.41–0.46)	(0.40–0.48)	(0.34–0.43)	(0.40–0.48)	(0.33–0.33)	(0.38–0.47)	(0.39–0.50)
**MAE**	0.62	0.65	0.77	0.66	1.00	0.75	0.65
	(0.60–0.65)	(0.61–0.68)	(0.70–0.83)	(0.60–0.71)	(1.00–1.00)	(0.69–0.81)	(0.59–0.71)
**MCC**	0.17	0.17	0.08	0.17	0.00	0.14	0.19
	(0.14–0.20)	(0.11–0.22)	(0.01–0.14)	(0.10–0.23)	(0.00–0.00)	(0.07–0.20)	(0.10–0.25)
**CK**	0.15	0.17	0.07	0.16	0.00	0.13	0.19
	(0.12–0.19)	(0.11–0.22)	(0.01–0.14)	(0.10–0.22)	(0.00–0.00)	(0.07–0.20)	(0.09–0.25)

a Metrics include accuracy (Acc), mean absolute error (MAE), Matthews correlation coefficient (MCC), and Cohen’s kappa (CK). They are reported as median (5%–95% percentile confidence interval).

The confusion matrix for the METABRIC dataset (Table 8) reveals a consistent bias toward the intermediate class (class 1) across all three groups. Among the 105 samples of each class, per-class accuracies are 39% for class 0, 63% for class 1, and 42% for class 2, with a large proportion of class 0 and class 2 samples being misclassified as class 1 (51 and 54 samples, respectively). Importantly, since the dataset was partitioned into three equally sized groups, this bias cannot be attributed to class imbalance. One possible explanation may reside in the ensemble architecture itself: as each BMC relies on a single gene pair, the prognostic signal relevant to a given patient may only be captured by a single BMC (specific to the patient), while the two others receiving little discriminative signal, could tend to default toward the intermediate class. Majority voting would then amplify this default, diluting the signal of the informative BMC and systematically pulling predictions toward class 1.

**Table 8:** Confusion matrix for the METABRIC dataset.^a^

		Predicted label		
		Short RFS	Mid RFS	Long RFS
True label	Short RFS	41	51	13
	Mid RFS	20	66	19
	Long RFS	7	54	44

a Rows represent true labels and columns represent predicted labels, with classes 0, 1, and 2 corresponding to low, intermediate, and high-risk groups, respectively.

Interestingly, the Kaplan–Meier analysis revealed that the ensembleMBMC effectively distinguished between the different RFS groups. This discrepancy underscored the challenge of using traditional metrics to accurately assess model performance in a multiclass biomedical context. One possible reason for this disparity could be that the metrics did not adequately capture the temporal dynamics of the data that were reflected in the ensembleMBMC.

We note that the analysis was restricted to patients with observed recurrence as the ordinal outcome requires all patients to have experienced the event. This selection criterion introduces conditioning bias and limits the applicability of results to recurrent cases only, restraining generalization to the full METABRIC cohort.

#### 3.3.2 Biological insights by looking at the top-performing pairs

We then studied the pairs obtained with a parameter k=5. Proceeding in the same way as for the ensembleMBMC in Section 3.3.1, we predicted the testing data on the eight identified top-performing pairs, trained with the training data, and separated them into three groups for the Kaplan–Meier curves (see Supplementary Material, available as supplementary data at *Bioinformatics Advances* online). We also used a logarithmic rank test to statistically compare survival curves, with a significance threshold of $\alpha =\frac{0.05}{8\times 3}=0.002$. The first observation was that, for each pair, the positions of the curves to one another were consistent with the predictions. Of the eight pairs, none were significantly different between the three prediction groups. However, for each pair, only one test between two groups was not significant, either short versus mid or long versus mid RFS. This indicates that samples predicted to have medium RFS sometimes had recurrence profiles similar to those with short RFS and other times to those with long RFS. Consequently, individually, the gene pairs did not significantly distinguish the three RFS classes. Further investigation could help to understand which genes differentiate (or fail to differentiate) the mid RFS class. A detailed illustration, including the Kaplan–Meier curves for the eight top-pairs and the statistical tests, is provided in the Supplementary Material, available as supplementary data at *Bioinformatics Advances* online.

## 4 Discussion

We introduced MBMC, an interpretable monotonic ordinal classifier that identifies gene pairs whose joint expression is associated with ordered clinical outcomes. Additionally, MBMC can serve as a lightweight predictive model in resource-constrained settings or as an interpretable complement to higher-capacity methods when mechanistic understanding is required for clinical decision-making. Evaluated on three cancer types (glioblastoma, ovarian high-grade serous cancer; HGSC, and breast cancer), MBMC achieves predictive performance comparable with established methods while offering distinct advantages in interpretability and biological insight. Each MBMC classifier uses only two genes with monotonic decision boundaries, enabling visual inspection, pathway enrichment analysis, and hypothesis generation about gene-gene interactions. The approach may capture survival-associated patterns through pairwise relationships not detectable by univariate differential expression analysis. Methodological limitations include potential loss of low-variance but clinically relevant genes during MAD filtering, and the inability to handle censored data, unlike Kaplan–Meier methods as MBMC requires observed outcomes for all samples, limiting applicability when events have not occurred. Finally, while we demonstrated applicability across three distinct clinical settings with robust held-out evaluation within each cohort, cross-cohort validation within the same cancer type, and with carefully harmonized endpoints, preprocessing, and platform differences remains an important next step to assess generalizability across independent patient populations and different molecular profiling platforms.

For future work, although we used pathway enrichment analysis to analyze the biological significance of the identified gene expression pairs, further investigation could be performed to understand the underlying biology. For example, the resulting gene expression pairs can be represented as a network of edges that connect gene expression pairs, which may provide further higher-order insight. Advanced network analysis techniques can be applied to uncover patterns and biological signals within this network. In addition, future studies can explore the possibility of stratifying patients according to the gene expression pairs that are the most predictive of the outcomes, potentially leading to more personalized and effective treatment strategies.

Extending the pairwise approach to higher orders (three or more genes) seems like a tempting idea but poses at least two serious problems. First, strongly increased computational complexity: while Stout (2013) provided regression algorithms for more than two features, their computational complexity increases strongly with their number. In addition, the number of feature subsets to be tested increases combinatorially. The second concern is overfitting: even if computational problems can be addressed, the large space of feasible solutions may lead to too many spurious findings that are impossible to distinguish from gene sets with a true monotonic relationship to the outcome.

## Supplementary Material

vbag143_Supplementary_Data

## Data Availability

The glioblastoma data are publicly available from the NCBI Gene Expression Omnibus under accession GSE53733. The METABRIC breast cancer data are publicly available via cBioPortal at the study page for brca_metabric. The high-grade serous ovarian carcinoma data from the DECIDER project were analyzed under project-specific access arrangements; availability of these data is subject to the data-sharing and ethical policies of the DECIDER consortium. Requests for access should be directed to the DECIDER project. The code used for this study is available at https://github.com/oceanefrqt/MBMC.

## References

[vbag143-B1] Cardoso JS , Sousa R. Measuring the performance of ordinal classification. Int J Patt Recogn Artif Intell 2011;25:1173–95. 10.1142/S0218001411009093

[vbag143-B2] Chen C-K. The classification of cancer stage microarray data. Comput Methods Programs Biomed 2012;108:1070–7. 10.1016/j.cmpb.2012.07.001 22925656

[vbag143-B3] Conde D , Fernández MA, Rueda C et al Isotonic boosting classification rules. Adv Data Anal Classif 2021;15:289–313. 10.1007/s11634-020-00404-9

[vbag143-B4] Curtis C , Shah SP, Chin S-F et al The genomic and transcriptomic architecture of 2,000 breast tumours reveals novel subgroups. Nature 2012;486:346–52. 22522925 10.1038/nature10983 PMC3440846

[vbag143-B5] Feng J , Zhao L, Fu L et al Kdelr3 overexpression as a novel prognostic and diagnostic biomarker in glioma: comprehensive bioinformatic analysis insights. Sci Rep 2024;14:30783. 10.1038/s41598-024-80991-1 39730475 PMC11681132

[vbag143-B6] Fourquet O , Krejca MS, Doerr C et al Towards the genome-scale discovery of bivariate monotonic classifiers. BMC Bioinformatics 2025;26:228. 10.1186/s12859-025-06253-7 40898061 PMC12403431

[vbag143-B7] Frank E , Hall M. A simple approach to ordinal classification. Lect Notes Comp Sci 2001;2167:145–56.

[vbag143-B8] Gaudette L , Japkowicz N. Evaluation methods for ordinal classification. Adv Artif Intell 2009;5549:207–10. 10.1007/978-3-642-01818-3_25

[vbag143-B9] Ghosh D. Incorporating monotonicity into the evaluation of a biomarker. Biostatistics 2007;8:402–13. 10.1093/biostatistics/kxl018 16905591

[vbag143-B10] Gutiérrez PA , García S. Current prospects on ordinal and monotonic classification. Prog Artif Intell 2016;5:171–9. 10.1007/s13748-016-0088-y

[vbag143-B11] Hakkinen A , Zhang K, Alkodsi A et al Prism: recovering cell-type-specific expression profiles from individual composite RNA-seq samples. Bioinformatics 2021;37:2882–8. 10.1093/bioinformatics/btab178 33720334 PMC8479664

[vbag143-B12] Howell DC. Median absolute deviation. Encyclopedia of Statistics in Behavioral Science. Hoboken, NJ: John Wiley & Sons, Ltd, 2005.

[vbag143-B13] Lahtinen A , Lavikka K, Virtanen A et al Evolutionary states and trajectories characterized by distinct pathways stratify patients with ovarian high grade serous carcinoma. Cancer Cell 2023;41:1103–17.e12. 10.1016/j.ccell.2023.04.017 37207655

[vbag143-B14] Luyckx M , Squifflet J-L, Bruger AM, et al Recurrent high grade serous ovarian cancer management. Ovarian Cancer. Brisbane: Exon Publications, 2022, 83–103. 36343139

[vbag143-B15] Nikolayeva I , Bost P, Casademont I et al A blood RNA signature detecting severe disease in young dengue patients at hospital arrival. J Infect Dis 2018;217:1690–8. 10.1093/infdis/jiy086 29490079 PMC5946912

[vbag143-B16] Pedregosa F , Varoquaux G, Gramfort A et al Scikit-learn: machine learning in python. J Mach Learn Res 2011;12:2825–30.

[vbag143-B17] Pereira B , Chin S-F, Rueda OM et al The somatic mutation profiles of 2,433 breast cancers refines their genomic and transcriptomic landscapes. Nat Commun 2016;7:11479. 10.1038/ncomms11479 27161491 PMC4866047

[vbag143-B18] Reifenberger G , Weber RG, Riehmer V et al; German Glioma Network. Molecular characterization of long-term survivors of glioblastoma using genome- and transcriptome-wide profiling. Int J Cancer 2014;135:1822–31. 10.1002/ijc.28836 24615357

[vbag143-B19] Stout QF. Isotonic regression via partitioning. Algorithmica 2013;66:93–112. 10.1007/s00453-012-9628-4

[vbag143-B20] Stroggilos R , Frantzi M, Zoidakis J et al Gene expression monotonicity across bladder cancer stages informs on the molecular pathogenesis and identifies a prognostic eight-gene signature. Cancers (Basel) 2022;14:2542. 10.3390/cancers14102542 35626146 PMC9140126

[vbag143-B21] Tian S. Identification of monotonically differentially expressed genes for non-small cell lung cancer. BMC Bioinformatics 2019;20:177. 10.1186/s12859-019-2775-8 30971213 PMC6458730

[vbag143-B22] Wang HW , Sun HJ, Chang TY et al Discovering monotonic stemness marker genes from time-series stem cell microarray data. BMC Genomics 2015;16:S2. 10.1186/1471-2164-16-S2-S2 PMC4331716 25708300

[vbag143-B23] Wolter M , Felsberg J, Malzkorn B et al Droplet digital PCR-based analyses for robust, rapid, and sensitive molecular diagnostics of gliomas. Acta Neuropathol Commun 2022;10:42. 10.1186/s40478-022-01335-6 35361262 PMC8973808

[vbag143-B24] Wu W , Jiang C, Zhu W et al Multi-omics analysis reveals the association between specific solute carrier proteins gene expression patterns and the immune suppressive microenvironment in glioma. J Cell Mol Med 2024;28:e18339. 10.1111/jcmm.18339 38687049 PMC11060081

[vbag143-B25] Yao Z , Zhang Q, Guo F et al Long noncoding RNA PCED1B-AS1 promotes the Warburg effect and tumorigenesis by upregulating HIF-1α in glioblastoma. Cell Transplant 2020;29:096368972090677. 10.1177/0963689720906777 PMC7444212 32326742

[vbag143-B26] Zhang L , He L, Lugano R et al IDH mutation status is associated with distinct vascular gene expression signatures in lower-grade gliomas. Neuro Oncol 2018;20:1505–16. 10.1093/neuonc/noy088 29846705 PMC6176806

