# Supplementary material for: Identification of monotonically classifying pairs of genes for ordinal disease outcomes
Source: Bioinform Adv. 2026 May 22;6(1):vbag143. doi: 10.1093/bioadv/vbag143 (PMC13238737; doi:10.1093/bioadv/vbag143)
Supplement: vbag143_Supplementary_Data [file vbag143_supplementary_data.pdf]

Identification of Monotonically Classifying Pairs of Genes for Ordinal Disease Outcomes: Supplementary Material

May 18, 2026

2. Methods

2.1. Data Sets

Glioblastoma Data Set

This data set was obtained using microarray technology from primary tumor samples of 70 patients with glioblastoma in the German Glioma Network. Among the patients are 23 long-term survivors ($>$ 36 months overall survival), 16 short-term survivors ($<$ 12 months overall survival), and 31 patients with intermediate overall survival, as defined in Reifenberger et al. [2014]. Only genes with high variability were selected, using the median absolute deviation (MAD) technique (Howell [2005]), with a threshold of 0.4, which results in 1,836 genes to analyze. Raw RNA sequencing data are publicly available on the National Center for Biotechnology Information Gene Expression Omnibus repository (`https://www.ncbi.nlm.nih.gov/geo/`) with the reference GSE53733. They were generated with the Affymetrix Human Genome U133 Plus 2.0 platform with over 50,000 probesets on chips. Only protein-coding genes were kept, resulting in around 16,000 genes. Multiple probes linked to the same gene were gathered and averaged into a single gene. Gene expression was normalized using the TMM (Trimmed Mean of M-values) method followed by a log1p transformation to stabilize the variance and ensure that the data are on a suitable scale for downstream analysis.

Ovarian High-Grade Serous Carcinoma Data Set

The DECIDER project (`https://www.deciderproject.eu`) has produced a RNAseq data set of high-grade serous ovarian carcinoma (HGSC) samples, featuring various sample types, including primary tumors, intra-abdominal lesions, and ascites, from a well-characterized cohort (`https://clinicaltrials.`

gov/study/NCT04846933?tab=table). Data were processed as described in Lahtinen et al. [2023]. The patients in the cohort underwent surgery, allowing tissue sample collection, followed by platinum-based chemotherapy. Our study focused on the prediction of the time between the last chemotherapy cycle and relapse observation, called the platinum-free interval (PFI), and was based on samples collected before treatment. To account for the heterogeneity of the samples, the PRISM algorithm (Hakkinen et al. [2021]) was used to deconvolute the bulk RNAseq data into cancer-, immune- and stromal-specific expression profiles. For this study, only cancer-specific profiles were used in the analysis. To avoid redundancy, the sample with the highest tumor purity score, as estimated by PRISM, was selected for patients with multiple samples. Finally, gene expression data were normalized using the TMM method followed by a log1p transformation. Genes were filtered using the median absolute deviation technique, with thresholds of 1.6, 1.4, and 1.8 for primary tumors, intra-abdominal lesions, and ascites, respectively. The different sub-data-sets are described in Table 2 of Section 2.1.2. The classes were determined according to the common clinical classification that considers patients resistant to platinum-based chemotherapy when their PFI is less than 6 months, semi-sensitive when their PFI is between 6 and 12 months, and sensitive when the PFI is greater than one year (Luyckx et al. [2022]).

Breast Cancer Data Set

METABRIC (Curtis et al. [2012], Pereira et al. [2016]) is a well-known data set encompassing several types of genetic and genomic data, of more than 2,000 patients (https://www.cbioportal.org/study/summary?id=brca_metabric). DNA and RNA were extracted from each sample in a matched manner and then analyzed for copy number and genotype variations using the Affymetrix SNP 6.0 platform, while transcriptional profiling was performed on the Illumina HT-12 v3 platform. The transcriptomic data were already normalized. We used the median absolute deviation (MAD) technique, with a threshold of 0.7, resulting in 1,708 genes. Our objective was to predict the relapse-free status (RFS) of the patients. Therefore, we only kept patients who had a recurrence, which corresponded to 630 patients. We took the whole range of RFS and separated them into three classes that contained the same number of samples.

2.2. Algorithmic Framework

2.2.1. Ordinal Classification

The performance of ordinal classification can be evaluated according to different metrics. Among the most common are (Cardoso and Sousa [2011] and Gaudette and Japkowicz [2009]):

- Accuracy (Acc): Ratio between the number of correct predictions and the total number of predictions.
- Accuracy within n: Proportion of predictions that are within a certain distance (n) of the actual class label.
- Mean Absolute Error (MAE): Mean difference between the predicted and actual class labels.
- Mean Squared Error (MSE): Similar to MAE, but it squares the differences between predicted and actual class labels.
- Cohen's Kappa (κ): statistical measure that evaluates the agreement between predictions actual class labels, correcting for chance agreement.
- Spearman's Rank Correlation Coefficient: Correlation between the predicted and actual class labels, taking into account the ordinal nature of the classes.
- Matthews Correlation Coefficient (MCC): Correlation coefficient using true positives, true negatives, false positives, and false negatives.

All of these metrics either do not take into account the inherent class order or assume that the classes have a fixed, equal spacing between them (Cardoso and Sousa [2011], Gaudette and Japkowicz [2009]). For our study, we have chosen to work with the following metrics: MAE, Acc, κ, and MCC.

We note that among all the above metrics, MAE is one of the most robust common metrics for ordinal classification (Gaudette and Japkowicz [2009]) and fits the flexible frame of ordinal classification. This is why we also use it in our approach. Moreover, we note that the way that the classes are assigned to numbers can have an impact. In order to avoid bias toward some classes, we thus assume in the following use cases that there is an equal absolute distance (of 1) between neighboring classes.

2.2.3. Multi-class Bivariate Monotonic Classifier (MBMC)

Before settling on majority voting as the aggregation scheme to be used in this study, we compared majority voting with median class prediction, an alternative that is often considered more natural for ordinal outputs. On two independent datasets, the performance difference was negligible: Accuracy and MAE were either identical or differed by less than 0.07 MAE units, and MCC differed by less than 0.004 (see Table 1). This confirms that the two strategies are empirically equivalent in this setting. Other aggregation schemes remain possible extensions of this work.

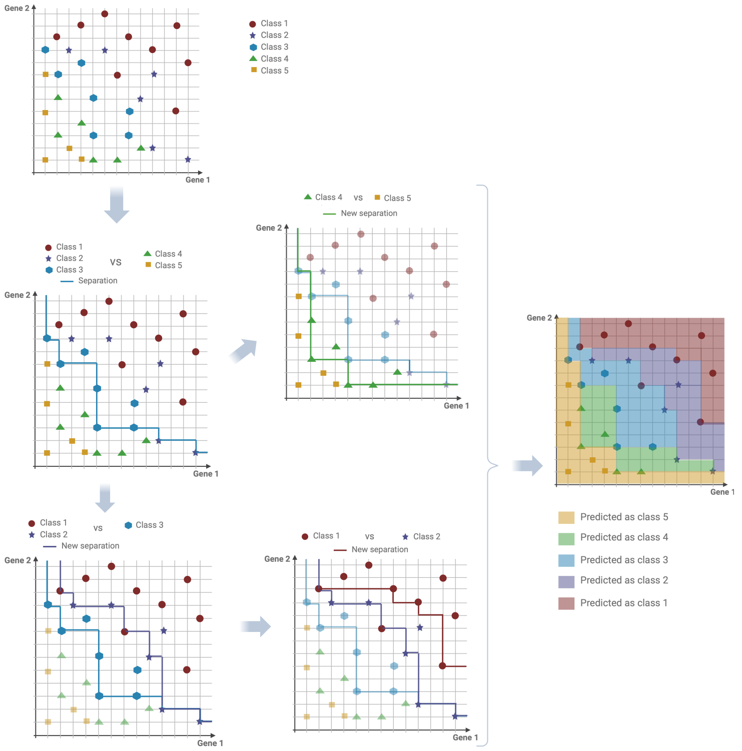

Figure 1: A step-by-step illustration of building a simple MBMC with 5 classes, showcasing the iterative process of creating separation functions and the final classifier, as described in Nikolayeva et al. (2018).

3. Empirical Evaluation

This section completes the results already provided in the Main paper. The tables providing the performance evaluation of different models, including the median of the performances across the top pairs, contain quartile intervals for the top pairs, allowing for a better representation of the performance of MBMCs.

Moreover, a comparative visualization of some pairs enables us to see the patterns captured by MBMCs versus other classical models.

Furthermore, when considering the top-performing pairs as a group, functional enrichment analyzes help uncover pathways that are enriched among these pairs. Functional enrichment analysis was performed using *enrichr* from the **gseapy** library (Fang et al. [2022]) and the three databases MSigDB Hallmark, MSigDB Oncogenic Signatures, and PID. For each data set, genes after filtering were used as a background for enrichr. The pairs were grouped according to their behavior and the orientation of the relationship. Orientation 1 corresponds to the group of pairs for which both gene expressions

Table 1: Performance comparison of majority and median voting strategies across breast cancer and glioblastoma datasets.

**Dataset**	**Strategy**	**Accuracy**	**MAE**	**MCC**
Breast Cancer	Majority voting	0.479	0.584	0.231
	Median voting	0.479	0.584	0.232
Glioblastoma	Majority voting	0.571	0.500	0.369
	Median voting	0.571	0.429	0.365

Table 2: Jaccard similarity of retained genes of ensembleMBMC built for different MAD thresholds filtering.

	**0.35**	**0.37**	**0.39**	**0.41**	**0.43**	**0.45**
**0.35**	1.00	0.05	0.25	0.05	0.00	0.00
**0.37**	0.05	1.00	0.18	0.05	0.05	0.05
**0.39**	0.25	0.18	1.00	0.11	0.00	0.11
**0.41**	0.05	0.05	0.11	1.00	0.05	0.05
**0.43**	0.00	0.05	0.00	0.05	1.00	0.00
**0.45**	0.00	0.05	0.11	0.05	0.00	1.00

increase with the classes. Similarly, Orientation 2 is for pairs whose gene expressions decrease with the classes. And the last group, Orientation 3, is for the mixed signals (one gene increasing and the other decreasing).

3.1. Results on Glioblastoma Data Set

To assess the sensitivity of our method to the MAD filtering threshold, we systematically varied it from 0.35 to 0.45 across the glioblastoma dataset. We evaluated both the stability of the selected features and downstream classification performance. At the gene level (Table 2), consecutive thresholds retain partially overlapping gene sets (Jaccard similarity 0.05˘0.25), suggesting

Table 3: Jaccard similarity of retained gene pairs of ensembleMBMC built for different MAD thresholds filtering.

	**0.35**	**0.37**	**0.39**	**0.41**	**0.43**	**0.45**
**0.35**	1.00	0.00	0.00	0.00	0.00	0.00
**0.37**	0.00	1.00	0.11	0.00	0.00	0.00
**0.39**	0.00	0.11	1.00	0.00	0.00	0.00
**0.41**	0.00	0.00	0.00	1.00	0.00	0.00
**0.43**	0.00	0.00	0.00	0.00	1.00	0.00
**0.45**	0.00	0.00	0.00	0.00	0.00	1.00

Table 4: Classification performance of the ensembleMBMC on the test set for different MAD thresholds filtering.

MAD threshold	Acc	MCC	MAE
0.35	0.500	0.202	0.571
0.37	0.571	0.320	0.500
0.39	0.500	0.262	0.500
0.41	0.500	0.209	0.571
0.43	0.643	0.458	0.429
0.45	0.357	−0.052	0.714

that the individual features selected are not wholly arbitrary. However, gene-pair overlap (Table 3), which reflects the relational structure the classifier actually exploits, is nearly zero across all threshold pairs (Jaccard ≤ 0.05 at preselection), indicating that the inferred monotonic relationships are highly threshold-dependent even when the constituent genes are shared. This dissociation between gene-level and pair-level stability underscores that MAD thresholding does not merely reduce dimensionality uniformly: it reshapes the interaction landscape the model operates on. In terms of classification performance (Table 4), a threshold of 0.37 consistently outperforms alternatives, achieving MCC up to 0.57 and accuracy up to 0.71, while thresholds at 0.45 yield near-random or negative MCC.

Table 5: Confusion matrix for the glioblastoma data set. Rows represent true labels and columns represent predicted labels, with classes 0, 1, and 2 corresponding to the respective risk groups.

		**Predicted label**		
		**STS**	**ITS**	**LTS**
	**STS**	2	1	0
**True label**	**ITS**	1	5	0
	**LTS**	1	3	1

As seen in Table 5, The confusion matrix for the glioblastoma dataset reveals an overall accuracy of 57.1% (8/14 samples), highlighting specific areas of class ambiguity. While the model demonstrates robust performance for mid-term survivors with a recall of 83%, it reveals a significant diagnostic bias: long-term survivors are frequently misclassified, with 60% of them being incorrectly labeled as intermediate-term survivors. It should be noted that in the event of a tie in majority voting, the final decision is made in favor of the worst outcome, which might be leading to this significant bias. Overall, this confusion matrix shows that the "middle" class acts as a primary point of confusion, absorbing errors from both the short and long-

term survivors. A thorough analysis of what led the short-term patient to be classified as intermediate could help explain the inaccurate prediction and allow the model to be recalibrated to avoid such errors.

Figure 2 illustrates two of the pairs of genes that perform the best as identified by the MBMC, as well as the associated classifiers, constructed using the competing algorithms. The scattered dots represent the training data points and the colored background corresponds to the classifier built from the data[1], where each color denotes one of the three classes. These classes are associated with survival outcomes, ranging from the lowest survival rate (Class 0) to the highest survival rate (Class 2), with Class 1 representing an intermediate survival rate. This visualization helps to easily understand the behaviors of the two gene expressions and their relations with the ordinal outcomes.

From a comparable visual perspective, our model stands out for its clear and identifiable pattern, allowing intuitive understanding and generalization of the underlying trend. Although it may not be the most precise, its simplicity enables the formulation of realistic hypotheses that can be applied in real-world scenarios. Notably, our model's pattern is more generalizable and interpretable than that of a decision tree, which, despite similarities, can be limited by its rigid structure. In contrast, logistic regression, while capable of capturing the monotone trend, often does so with less nuance and detail, failing to provide the same level of insight as our model. However, traditional algorithms are trained on many more than just two genes. A visualization of this order therefore requires a dimensional reduction, such as a PCA, making interpretation all the more difficult, in stark contrast to MBMC. Of course, this visual comparison needs to be qualified. Classical classification models are trained on all genes, not just pairs.

According to the performance ranking (Table 6), the top-performing algorithms for the glioblastoma data set were logistic regression and SVM_{rbf} which achieved the highest rankings in several metrics. In comparison, the MBMC algorithms, with the selection parameters set at 5, 10, and 20, did not achieve the highest ranking but still demonstrated competitive performance (Table 6), generally outperforming algorithms such as Gaussian processes and decision trees. Although the performance of the MBMC algorithms did not exceed the other top-performing models, they maintained consistent performance across the metrics. In particular, the performance gap between the MBMC algorithms and the models that performed worst, such as Gaussian processes, was substantial, indicating that the MBMC approach remains a viable option for the analysis of the glioblastoma data set.

Among the selected pairs, the identification of a signature of gene pairs and a corresponding **ensemble model (ensembleMBMC)** offers a promis-

[1]Note that these are the models obtained when trained on all the data, but the MAE-CVE corresponds to the MAE calculated with the 5-fold CV.

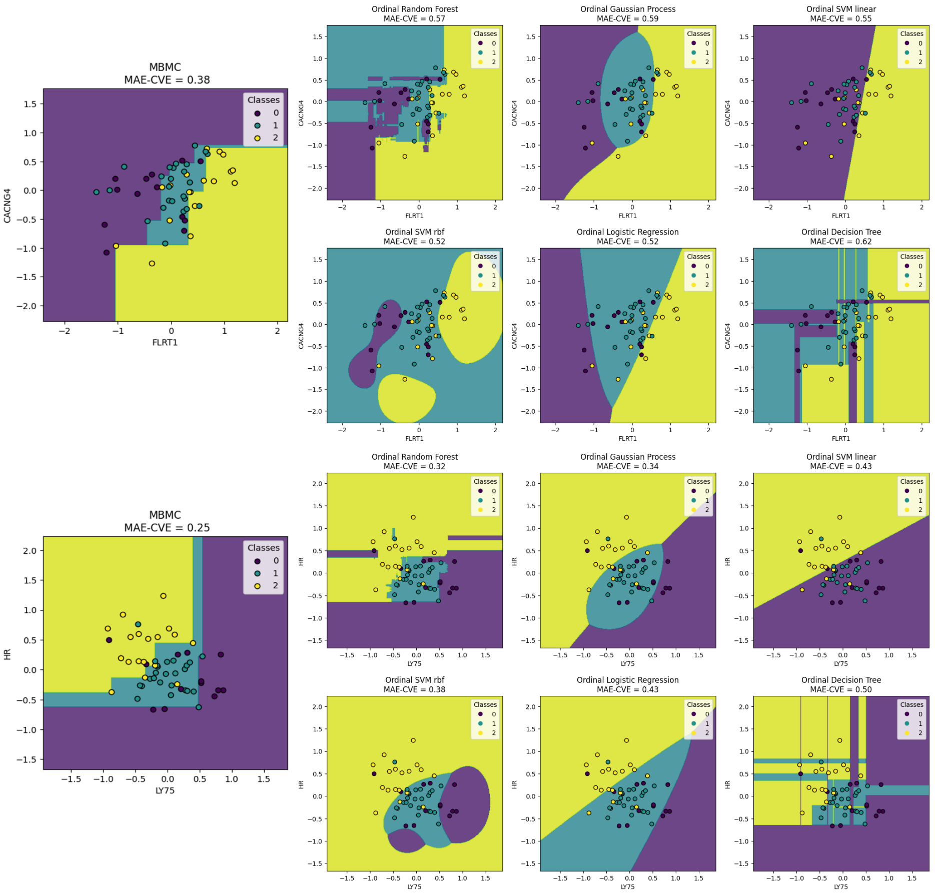

Figure 2: Comparison of the visual representation of the classifiers for two of the best pairs of genes constructed on glioblastoma training data (described in the Main Article). The dots represent the training data points and the colored background corresponds to the classifier built from these data points. The two models one on the left are from MBMC, the other models are from the competing approaches (described in the Main Article).

ing approach to improve predictive performance. By focusing on a compact signature, ideally comprising around 10 genes or fewer, we can use the results of the first scenario. To construct our ensemble classifier, we selected five non-redundant gene pairs that do not share common genes (Fig. 1 of main paper). We trained this ensemble classifier on the training data. Upon testing this ensemble classifier on the testing data, we achieved a **MAE of 0.43**, which surpasses the performance of all other methods explored. This result shows that multiple pairs of high-performing genes can be combined to form strong predictors.

After pathway enrichment analysis, it was determined that the gene pairs in Orientation 1 were enriched for pathways that are typically associated

Table 6: Performance evaluation of different models, with metrics including Mean Absolute Error (MAE), Accuracy (Acc), Matthews Correlation Coefficient (MCC), and Cohen's Kappa for the glioblastoma data set. It also includes in parenthesis the ranking of models based on their performance, with the top-performing model ranked 1st and subsequent models ranked accordingly.

	Algorithms									
	MBMC-5	MBMC-10	MBMC-20	ensembleMBMC	RF	DT	LR	GP	SVM_{linear}	SVM_{rbf}
MAE	0.57 (4)	0.57 (4)	0.64 (5)	0.43 (**1**)	0.54 (3)	0.64 (5)	0.50 (2)	1.14 (6)	0.64 (5)	0.50 (2)
MCC	0.22 (5)	0.22 (5)	0.22 (5)	0.37 (**1**)	0.36 (2)	0.36 (2)	0.37 (**1**)	0.00 (6)	0.35 (3)	0.26 (4)
κ	0.20 (5)	0.18 (6)	0.20 (5)	0.31 (2)	0.28 (4)	0.31 (2)	0.29 (3)	0.00 (8)	0.34 (**1**)	0.13 (7)
Acc	0.50 (3)	0.50 (3)	0.50 (3)	0.57 (**1**)	0.57 (**1**)	0.54 (2)	0.57 (**1**)	0.21 (4)	0.57 (**1**)	0.50 (3)

with breast cancer, including early estrogen response, SRC UP.V1 DN, LTE2 UP.V1 DN, and EGFR UP.V1 DN. It is possible that the underlying biological processes regulated by these pathways, such as cell proliferation and survival, are also relevant to glioblastoma. For example, the SRC pathway is known to play a role in cell migration and invasion, which are also hallmarks of glioblastoma. Similarly, the EGFR pathway is often dysregulated in glioblastoma, leading to increased cell proliferation and survival. Orientation 2 pairs were enriched for the epithelial-mesenchymal transition (EMT) and PDGF UP.V1 DN. The EMT pathway is a process linked to tumor progression and invasiveness, which correlates with a poorer prognosis in glioblastoma. The PDGF UP.V1 DN pathway involves genes downregulated in neuroblastoma cells in response to Platelet-Derived Growth Factor (PDGF) stimulation. The last group of gene pairs is enriched for SNF5 DN.V1 DN, ATF2 UP.V1 DN, BMI1 DN MEL18 DN.V1 DN, and RELA DN.V1 UP. These pathways involve genes down-regulated in response to perturbations, such as the knockout of SNF5, a tumor suppressor gene, or the over-expression of ATF2, a transcription factor involved in cell growth and survival. The BMI1 DN MEL18 DN.V1 DN pathway, which is associated with the down-regulation of genes involved in stem cell self-renewal, is also notable, as it suggests a potential link between glioblastoma and cancer stem cell biology. The RELA DN.V1 UP pathway, which involves genes upregulated after the knockdown of the NF-κB subunit RELA, may indicate a role for inflammatory signaling in glioblastoma.

3.2. Results on Ovarian High-Grade Serous Carcinoma

3.2.1. Differences Between Sample Types

The results of the three scenarios, including the number of pairs obtained and the number of distinct genes that comprise them, are summarized in Table 7. For the three scenarios, the intra-abdominal data set is the one for which a higher number of top pairs is identified. The other two data sets are quite similar in terms of numbers.

Table 7: Summary of the number of top pairs and genes identified across the different sample types in the three scenarios.

		**Primary Sites**	**Ascites**	**Intra-Abdominal**
**Number of top pairs**	MBMC-5	12	12	23
	MBMC-10	24	37	58
	MBMC-20	102	81	198
**Number of genes among the top pairs**	MBMC-5	20	17	26
	MBMC-10	36	50	63
	MBMC-20	115	98	175

A statistical test was performed to determine whether the observed overlap of genes between two top pairs groups (Table 8) was significantly greater than what would be expected by random chance. The top pairs from the primary sites and the intra-abdominal sites overlapped significantly more than by pure chance (p-value of 2e−5), but not in the two other comparisons between sites. Interesting, ascites also distinguish themselves biologically from the two other tissue sites, through their character as *fluid* tissue, and their specific oncological classification as a distant metastasis.

Table 8: Overlapping genes between the top pairs in the subtypes.

	**Primary Sites**	**Ascites**	**Intra-Abdominal**
**Primary Sites**	115	5	21
**Ascites**		98	9
**Intra-Abdominal**			175

To gain a better understanding of the performance of the MBMC algorithms across different gene pairs and subtypes of samples, the MAE values were calculated for all possible genes. pairs. Figure 3 visualize the distribution of performance. The MAE distributions of every pair for the three subtypes of samples were close to normal distributions. The distribution of MAE values for ascites was more spread out compared to the other two subtypes, suggesting that there was more variability. The distribution of MAE

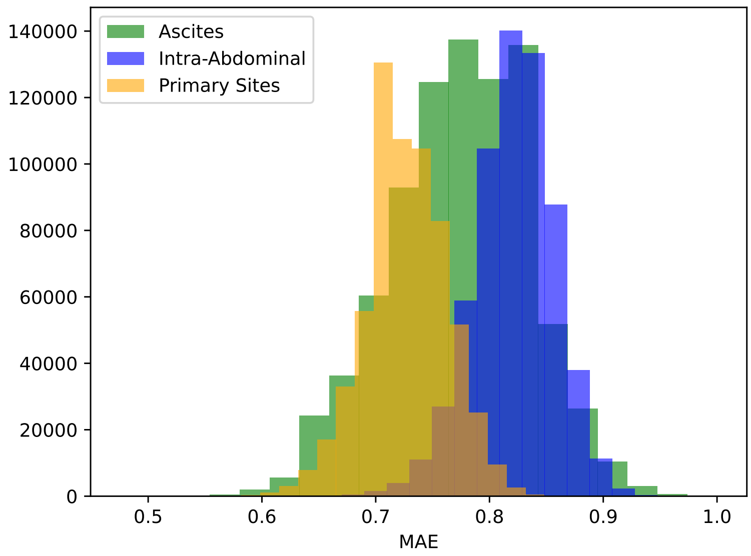

Figure 3: Distribution of mean absolute error values for all possible gene pairs across different subtypes of samples.

values for intra-abdominal metastasis was more peaked and has a shorter tail, indicating that the MAE values was more concentrated around a central value. The distribution of MAE values for primary sites was similar to that of intra-abdominal metastasis, but skewed toward lower MAE values.

To assess the significance of the observed MAE value, we determined whether the MAE of the top pairs was significantly better than what would be expected by chance. First, the class labels were randomly permuted. Then, 1,000 pairs of genes were randomly selected and their MAE values calculated. The frequency with which the randomized MAE value was less than or equal to the observed MAE value is used as a p-value. For the three data sets, this test results in p-values lower than 0.05, implying statistical significance at this level.

Analyzing the results, we observe the following trends:

- **Primary sites**: The decision tree achieved the best performance (MAE $= 0.59$), while the MBMC algorithms remained competitive, with MAE values between 0.67 and 0.70. Their performance was clearly superior to that of Gaussian Process and $\text{SVM}_{\text{linear}}$, indicating that the MBMC models offer a solid alternative despite not being the best performers.

- **Ascites**: MBMC-20 performed best among the MBMC scenarios, achieving results comparable to the random forest model. MBMC-5 and MBMC-10 also performed consistently well, while decision tree, logistic regression, and $\text{SVM}_{\text{linear}}$ showed much higher errors. In general, MBMC algorithms yielded promising predictive performance for

ascitic samples.

- **Intra-abdominal metastasis**: The decision tree again led the performance, closely followed by random forest and logistic regression. The MBMC algorithms showed slightly higher MAE values, but remained on par with Gaussian Process and SVM_{linear}, confirming their reasonable performance even in this more complex subtype.

For the three subtypes, enrichment analysis did not yield significant results, suggesting that identified gene pairs may not be sufficient to uncover the underlying biological mechanisms. A way to overcome this could be to consider a larger number of gene pairs. Moreover, there might be other possible reasons for this inconclusive enrichment analysis. One possibility is that higher-order interactions between genes are at play, which cannot be captured with only two genes. It is also possible that the identified gene pairs are involved in unknown or uncharacterized pathways that are not represented in the used databases. The relatively small number of patients in the study may also contribute to the lack of significant findings, as larger sample sizes are often required to detect subtle but significant differences. Finally, MBMCs enforce monotonicity relationships between genes and the PFI, which is a big assumption about the relationship.

Although pathway enrichment analysis had not produced statistically significant results in our study, examining individual gene pairs may still provide valuable information (Figure 4).

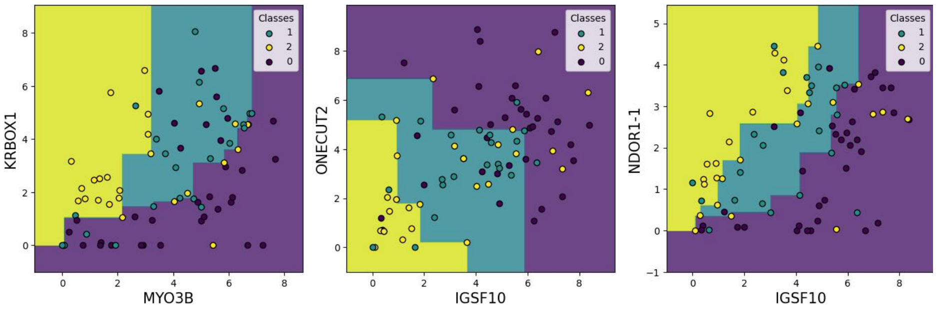

Figure 4: Visualization of three of the top-performing gene pairs, with the MBMC constructed on training data of ascites samples.

3.3. Results on Breast Cancer

In Table 11, we observe that accuracy alone is misleading under class imbalance. At the extreme 15/10/75 split, the accuracy reaches 0.75, but the confusion matrix reveals that the model collapses to predicting High for virtually all samples, a consequence of the class that makes up 75% of the

test set. The MCC of 0.08 at this threshold correctly identifies this as quasi-random discrimination. MCC is stable across less imbalanced thresholds. For the three intermediate configurations (20/20/60, 20/30/50, 25/25/50), MCC varies only between 0.24 and 0.26, demonstrating that the discriminative ability of the method is robust to the exact choice of cut-off. At 25/42/33, where the Mid group represents 42% of the samples, the MCC drops to 0.12, as the expanded middle class becomes increasingly difficult to distinguish from adjacent groups. This is an expected consequence of an ill-defined intermediate category, rather than a limitation of the model. MAE treats the three classes as ordinal and penalizes predictions that are further from the true class; however, like accuracy, it is inflated under class imbalance. In summary, results are consistent as long as there is not too great a difference in the number of samples between classes (one class containing 75% or more of the samples.

Table 9: Performance evaluation of different models, with metrics including Mean Absolute Error (MAE), Accuracy (Acc), Matthews Correlation Coefficient (MCC), and Cohen's Kappa, accross the three subtypes of samples, as well as the ranking of models based on their performance, with the top-performing model ranked 1st and subsequent models ranked accordingly, in parenthesis.

		Algorithms								
		MBMC-5	*MBMC-10*	*MBMC-20*	*RF*	*DT*	*LR*	*GP*	SVM_{linear}	SVM_{rbf}
Primary sites	MAE	0.67 (4)	0.70 (6)	0.69 (5)	0.61 (2)	0.59 (**1**)	0.72 (7)	1.28 (9)	0.81 (8)	0.62 (3)
	MCC	0.15 (5)	0.08 (6)	0.06 (7)	0.23 (3)	0.27 (**1**)	0.15 (4)	0.00 (9)	0.05 (8)	0.24 (2)
	κ	0.14 (5)	0.07 (6)	0.05 (8)	0.18 (2)	0.26 (**1**)	0.15 (4)	0.00 (9)	0.05 (7)	0.18 (3)
	Acc	0.47 (4)	0.44 (6)	0.44 (6)	0.55 (2)	0.52 (3)	0.47 (4)	0.22 (9)	0.41 (8)	0.56 (**1**)
Ascites	MAE	0.74 (3)	0.76 (4)	0.71 (**1**)	0.76 (4)	1.02 (7)	1.14 (8)	0.86 (6)	1.19 (9)	0.71 (3)
	MCC	0.05 (3)	0.02 (4)	0.08 (**1**)	0.07 (2)	-0.08 (7)	-0.30 (9)	0.00 (5)	-0.19 (8)	0.00 (5)
	κ	0.03 (3)	0.02 (4)	0.08 (**1**)	0.07 (2)	-0.08 (7)	-0.30 (9)	0.00 (5)	-0.18 (8)	0.00 (5)
	Acc	0.33 (4)	0.33 (4)	0.38 (2)	0.38 (3)	0.29 (6)	0.14 (9)	0.43 (**1**)	0.24 (8)	0.29 (6)
Intra-abdominal	MAE	0.95 (7)	0.92 (5)	0.92 (5)	0.90 (3)	0.74 (**1**)	0.85 (2)	1.05 (9)	1.00 (8)	0.90 (3)
	MCC	-0.04 (8)	-0.02 (6)	-0.02 (5)	0.04 (2)	0.10 (**1**)	0.03 (3)	0.00 (4)	-0.03 (7)	-0.04 (9)
	κ	-0.03 (8)	-0.02 (6)	-0.02 (5)	0.04 (2)	0.10 (**1**)	0.03 (3)	0.00 (4)	-0.03 (7)	-0.03 (9)
	Acc	0.31 (6)	0.31 (6)	0.31 (6)	0.38 (2)	0.40 (**1**)	0.36 (3)	0.36 (3)	0.36 (3)	0.28 (9)

Table 10: Performance evaluation of different models, with metrics including Mean Absolute Error (MAE), Accuracy (Acc), Matthews Correlation Coefficient (MCC), and Cohen's Kappa for the breast cancer data set. It also includes in parenthesis the ranking of models based on their performance, with the top-performing model ranked 1st and subsequent models ranked accordingly.

	Algorithms						
	ensembleMBMC	RF	DT	LR	GP	SVM_{linear}	SVM_{rbf}
MAE	0.61 (2)	0.62 (3)	0.79 (6)	0.66 (4)	1 (7)	0.73 (5)	0.59 (**1**)
MCC	0.2 (**1**)	0.19 (2)	0.07 (5)	0.18 (3)	0.00 (6)	0.13 (4)	0.19 (2)
κ	0.2 (**1**)	0.19 (2)	0.06 (6)	0.18 (3)	0.00 (7)	0.13 (5)	0.14 (4)
Acc	0.46 (**1**)	0.46 (**1**)	0.38 (5)	0.45 (2)	0.33 (6)	0.42 (4)	0.43 (3)

Table 11: Threshold sensitivity analysis on the METABRIC test set ($N = 315$). Thresholds define the percentile boundaries for Low, Mid, and High risk groups. Class sizes report the resulting number of samples per group. Metrics are computed on the test set; MCC is the primary metric given class imbalance.

**Threshold**	**Class size**			**MAE**	**Acc**	**MCC**
**(Low / Mid / High %)**	**Low**	**Mid**	**High**			
15 / 10 / 75	48	31	235	0.41	0.75	0.08
20 / 20 / 60	63	63	189	0.5	0.62	0.24
20 / 30 / 50	63	95	157	0.51	0.55	0.26
25 / 25 / 50	79	79	157	0.57	0.53	0.24
25 / 42 / 33	79	132	104	0.58	0.45	0.12

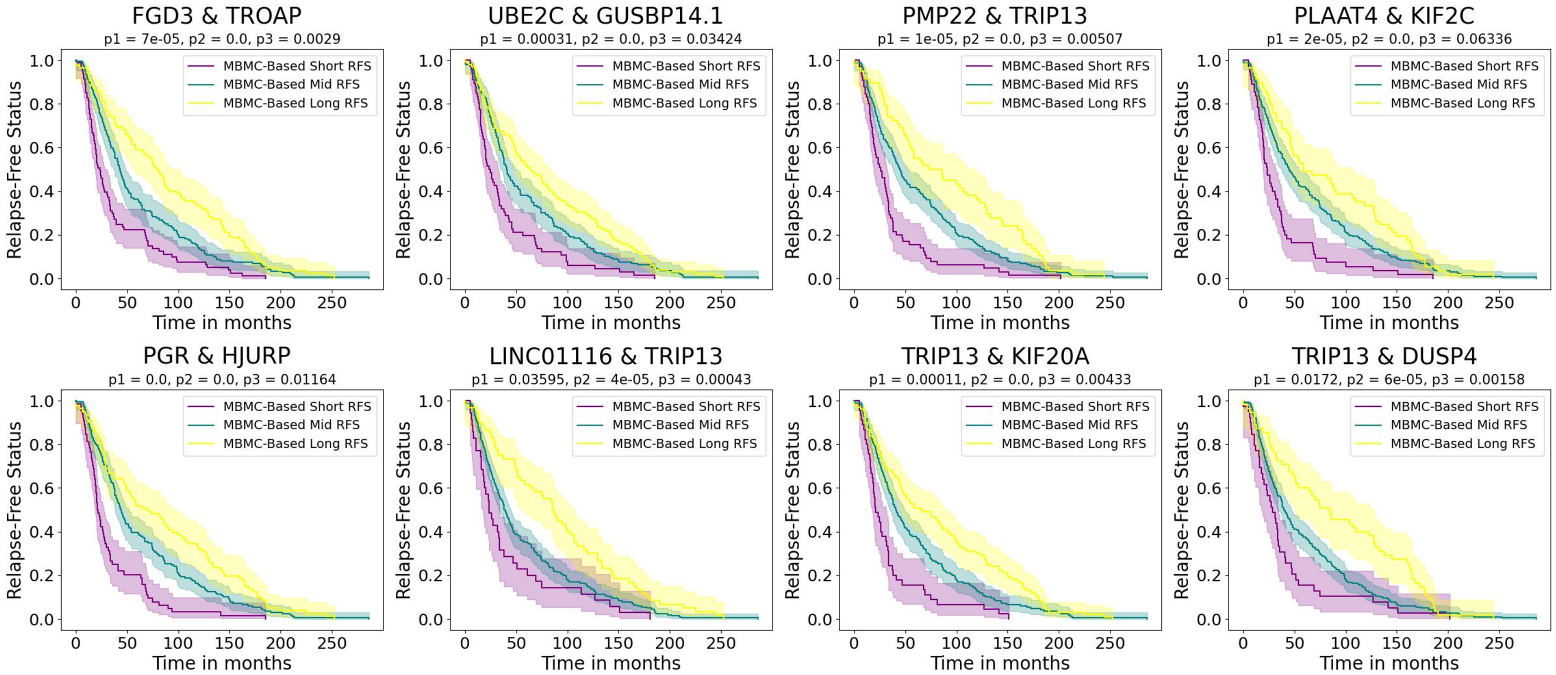

Figure 5: Illustration of the Kaplan–Meier curves for the eight top-performing pairs built on the training data of breast cancer, separating the testing data according to their predicted RFS. The colors are taken from those of the MBMCs visualization. The values of p1, p2 and p3 correspond to the p-values of the log-rank tests between short and mid RFS, short and long RFS, and mid and long RFS respectively. To be significant, p-values must be less than $\alpha = \frac{0.05}{8 \times 3} = 0.002$.

4. Technical Aspects of the Identification of the Top-Performing Gene Pairs

This section describes the algorithm with which the best MBMCs are identified and the mathematical property that allows fast preselection. It is a generalization of a preselection algorithm for BMCs (Fourquet et al. [2025]).

Theoretical Property

To introduce the preselection algorithm, we require a few definitions: Let S be a non-empty set of data points, and let P be a partition of S. Let C be a monotonic classifier over S and C_S an $L1$-optimal monotonic classifier over S.

For any set S', for any partition P' of S', and for any monotonic classifier C', let $E(C', S')$ denote the $L1$-error (L_1) of C' over S', and let $E(C', P')$ denote the L_1 of C' over P'. Then, $E(C', P') = \sum_{p \in P'} E(C', p)$ and $E(C', S') = \sum_{x \in S'} E(C', \{x\})$.

Moreover, the L_1 of C' over S' can be broken down into the sum of the L_1 of C' over each element x in S'. The elements in S' can be grouped into partitions p, so the L_1 of C' over S' is also equal to the sum over all parts $p$in P', and then summing over each element x within each partition. It can be simplified to the sum of the L_1 of C' over all partitions p. Ultimately, this means that the L_1 of C' over S' is equal to the L_1 of C' over P'.

Theorem 0.1. *For any non-empty set S and for any $p \in P$, it holds that $E(C_S, p) \leq E(C_{S \backslash p}, p)$.*

Proof.

1. For all parts p in P, the L_1 of C_S over S is lower than or equal to the L_1 of $C_{S \backslash p}$ over the subset S. This is because C_S is the optimal monotonic classifier over S in terms of $L1-$optimality.

2. For all parts p in P, the L_1 of C_S over $S \setminus p$ is greater than or equal to the L_1 of $C_{S \backslash p}$ over the subset $S \setminus p$. This is because $C_{S \backslash p}$ is the optimal monotonic classifier over $S \setminus p$ in terms of $L1-$optimality.

For all parts p in P, the L_1 of $C_{S \backslash p}$ over p is equal to the L_1 of $C_{S \backslash p}$ over S minus the L_1 of $C_{S \backslash p}$ over $S \setminus p$. According to Item 1, this L_1 is greater than or equal to the L_1 of C_S over S minus the L_1 of $C_{S \backslash p}$ over $S \setminus p$. Furthermore, according to Item 2, this is also greater than or equal to the L_1 of C_S over S minus the L_1 of C_S over $S \setminus p$, which is equal to the L_1 of C_S over p.

Knowing that, for all parts p of P, the L_1 of $C_{S \backslash p}$ over p is greater than or equal to the L_1 of C_S over p, by summing on all p, it holds that the sum of the L_1 of $C_{S \backslash p}$ over all parts p is greater than or equal to the sum of the L_1

of C_S over all partitions p, which is equal to the L_1 of C_S over P. Therefore, it is greater than or equal to L_1 of C_S over S. □

And $\sum_{p \in P} E(C_{S \setminus p}, p)$ is the classification error using cross-validation over P. Therefore, $\text{MAE}_{\text{CV}} \geq \text{MAE}_{\text{full}}$.

Preselection Algorithm

The property above allows efficient pair detection solely based on MAE_{full}, eliminating pairs that are bad due to a high MAE_{full}. The preselection algorithm to identify the top pairs was based on this property. It identified pairs with favorable MAE_{CV}. To achieve this, MAE_{full} was initially calculated for all pairs. Starting with pairs having the lowest MAE_{full}, their MAE_{CV} was computed. This process continued until the desired number of disjoint pairs—, i.e. pairs that do not contain the same genes,—was selected. Pairs with MAE_{CV} exceeding the maximum MAE_{full} among the selected pairs were discarded. To refine the selection threshold, MAE_{CV} were iteratively calculated for the remaining pairs, updating the maximum threshold whenever a new set of at least the target number of disjoint genes among the pairs was formed. This algorithm took as a parameter the number of disjoint genes which enable one to calibrate the outcome pairs.

The code for constructing MBMC and the preselection algorithm is available at `https://github.com/oceanefrqt/MBMC`

References

J. S. Cardoso and R. Sousa. Measuring the performance of ordinal classification. *International Journal of Pattern Recognition and Artificial Intelligence*, 25(08):1173–1195, 2011.

C. Curtis, S. P. Shah, S.-F. Chin, et al. The genomic and transcriptomic architecture of 2,000 breast tumours reveals novel subgroups. *Nature*, 486 (7403):346–352, 2012.

Z. Fang, X. Liu, and G. Peltz. Gseapy: a comprehensive package for performing gene set enrichment analysis in python. *Bioinformatics*, 2022.

O. Fourquet, M. S. Krejca, C. Doerr, et al. Towards the genome-scale discovery of bivariate monotonic classifiers. *BMC Bioinformatics*, 26:228, 2025.

L. Gaudette and N. Japkowicz. Evaluation methods for ordinal classification. In *Advances in Artificial Intelligence*, pages 207–210, 2009.

A. Hakkinen, K. Zhang, A. Alkodsi, et al. Prism: recovering cell-type-specific expression profiles from individual composite rna-seq samples. *Bioinformatics*, 37(18):2882–2888, 2021.

D. C. Howell. *Median Absolute Deviation.* John Wiley & Sons, Ltd, 2005.

T. Lahtinen et al. Evolutionary states and trajectories characterized by distinct pathways stratify patients with ovarian high grade serous carcinoma. *Cancer Cell*, 41(6):1103–1117.e12, 2023.

M. Luyckx, J.-L. Squifflet, A. M. Bruger, and J.-F. Baurain. Recurrent high grade serous ovarian cancer management. In *Ovarian Cancer*. 2022.

B. Pereira, S.-F. Chin, O. M. Rueda, et al. The somatic mutation profiles of 2,433 breast cancers refines their genomic and transcriptomic landscapes. *Nature Communications*, 7:11479, 2016.

G. Reifenberger, R. G. Weber, V. Riehmer, et al. Molecular characterization of long-term survivors of glioblastoma using genome- and transcriptome-wide profiling. *International Journal of Cancer*, 135(8):1822–1831, 2014.
